# Unplanned Readmissions due to Pleural Effusion Following the Fontan Operation

**DOI:** 10.1177/21501351261418295

**Published:** 2026-04-07

**Authors:** Jean M. Meneses, Mohan Pankhuri, Jake Beerel, Joseph B. Clark, John L. Myers, Allen R. Kunselman, Akif Ündar, Vincent P. R. Aluquin

**Affiliations:** 1 Penn State Hershey Pediatric Cardiovascular Research Center, 12311 Penn State College of Medicine, Penn State Health Children's Hospital, Hershey, PA, USA; 2 Department of Pediatrics, Penn State College of Medicine, Hershey, PA, USA; 3 Division of Pediatric Cardiology, Department of Pediatrics, McGovern Medical School, The University of Texas Health Science Center at Houston, Houston, TX, USA; 4 Division of Cardiovascular and Thoracic Surgery, Duke University Medical Center, Durham, NC, USA; 5 Department of Surgery, Penn State College of Medicine, Hershey, PA, USA; 6 Department of Public Health Sciences, Penn State College of Medicine, Hershey, PA, USA; 7 Department of Biomedical Engineering, College of Engineering, The Pennsylvania State University, University Park, PA, USA

**Keywords:** Fontan procedure, pleural effusion, readmission, pediatric cardiology, single ventricle physiology, postoperative complications, hospital readmission predictors, pre-Fontan hemodynamics, Fontan-associated complications

## Abstract

**Background:**

The Fontan operation is the final stage of palliation for single-ventricle patients. While operative survival has improved, pleural effusions remain a common postoperative complication, increasing morbidity and hospitalization. Our aim was to investigate the incidence and risk factors for unplanned readmissions due to post-Fontan pleural effusion.

**Methods:**

A retrospective review of all single ventricle patients who underwent the Fontan procedure from 2012–2023 was performed. This case-control study compared post-Fontan patients readmitted to the hospital due to pleural effusion with those who were not. Readmission was defined as rehospitalization for recurrent pleural effusion requiring intravenous diuretics and/or chest tube placement. Demographics, baseline clinical features, pre-Fontan hemodynamics, type of Fontan surgery, presence of fenestration, cardiac medications and doses, perioperative and post-discharge factors were analyzed between groups.

**Results:**

Sixty-one patients who were 42 ± 12.4 months of age underwent the Fontan operation. Of these, 31/61 (51%) had a diagnosis of hypoplastic left heart syndrome, 32/61 (52%) underwent extracardiac Fontan surgery, and 33/61 (54%) had a Fontan fenestration. Nine of 66 (14.7%) patients were readmitted for pleural effusion at a median of 7 days (IQR 5-15) post-discharge. Readmitted patients were older at Fontan (50.4 ± 13 vs 40.5 ± 11.8 months, *P* = .025), had increased chylothorax incidence (33.3% [3/9] vs 5.8% [3/52], *P* = .04), absent or restrictive fenestration at discharge (88.9% [8/9] vs 44.2% [23/52], *P* = .03) and were more likely to have pleural effusion at the first postoperative clinic visit (100% [9/9] vs 13.5% [7/52], *P* < .01). When comparing maximal diuretic usage, the readmitted patients required more diuretics (median 3 vs 2, *P* = .01). There were no significant differences in race, gender, weight at Fontan, cardiac anatomy, pre-Fontan hemodynamics, Fontan type, initial fenestration status, cardiopulmonary bypass duration time, supplemental oxygen duration, chest tube duration, number and doses of cardiac medications prior to and at Fontan discharge, or length of hospital stay. No patient required catheter-based intervention or surgery within 3 months post-Fontan discharge.

**Conclusion:**

Older age at Fontan, chylothorax, absent/restrictive fenestration at discharge, and detection of pleural effusion at the first postoperative clinic visit are risk factors for readmission due to pleural effusion. Earlier Fontan timing, optimizing diuretic management, and ensuring Fontan fenestration patency may reduce readmissions.

## Introduction

The Fontan procedure serves as the final stage of palliation in patients with single ventricle physiology. Despite advancements and modifications to the Fontan operation, there remain significant postoperative complications following the Fontan procedure that render this patient population vulnerable to experiencing diminished survival outcomes.^1,2^

The development of post-Fontan pleural effusions has been a commonly established complication associated with increases in morbidity, prolonged hospital stays, early mortality, hospital costs, and overall burden to patients and their families.^1,3,4^ Proposed factors such as elevated systemic venous pressures, impaired or abnormal lymphatic drainage, poor cardiopulmonary hemodynamics, absence of fenestration, prolonged cardiopulmonary bypass time, and chylothorax have traditionally been associated with post-Fontan pleural effusions.^5^

Several institutions have developed and adopted protocols designed to reduce the incidence and duration of persistent pleural effusions following the Fontan procedure. Such strategies include standardized Fontan management protocols,^3^ routine fenestration,^6^ optimized perioperative fluid management, early initiation of diuretic therapy,^3,7^ use of pulmonary vasodilators,^8^ and advanced lymphatic imaging and interventions.^9^ However, most studies assessing these approaches have been single-center, retrospective, and variable in how outcomes are defined. Differences in inclusion criteria, sample sizes, and reporting practices make it difficult to generate broad conclusions. This variability in institutional strategies reflects the current lack of clarity and consensus regarding universally accepted risk factors, despite reported improvements in outcomes.^10^ Furthermore, much of the existing literature has concentrated on index hospitalization, leaving less clarity around the drivers of recurrent pleural effusions and related readmissions.

In this study, we investigated the potential risk factors and clinical features of single ventricle patients with unplanned readmissions due to pleural effusions following the Fontan operation. By focusing on readmissions rather than index hospitalization, this study offers a unique perspective on management strategies for this specific morbidity post-Fontan. We hypothesized that the type of Fontan, presence of fenestration, chylothorax, and inadequate diuretic therapy are independently associated with an increased likelihood of unplanned readmission for pleural effusion.

## Patients and Methods

The study protocol was approved by the Institutional Review Board of Penn State College of Medicine. Informed consent was waived due to the study's retrospective nature.

### Study Design and Population

This retrospective case-control study included patients with single ventricle physiology who underwent Fontan completion at Penn State Health Children's Hospital between 2012 and 2023. Inclusion criteria required completed medical records encompassing demographic, preoperative, perioperative, and postoperative data. Patients without follow-up data at our institution were excluded. The primary outcome was hospital readmission following Fontan discharge due to clinically significant pleural effusion requiring intravenous diuretics and/or chest tube placement.

### Surgical Procedure

Fontan completion was generally performed at approximately 4 years of age but timing may have varied based on the pediatric cardiologist's clinical judgement. Indications for earlier intervention included progressive cyanosis, declining exercise tolerance, or evidence of polycythemia (hematocrit > 50). All patients underwent pre-Fontan cardiac catheterization to evaluate hemodynamics, define pulmonary arterial and inferior vena caval anatomy, and identify any structural abnormalities such as pulmonary artery (PA) stenosis, recoarctation of the aorta, venovenous or aortopulmonary collaterals, and pulmonary arteriovenous malformations. Interventional procedures were performed as indicated.

Decisions regarding fenestration and Fontan type were individualized based on anatomic considerations, preoperative hemodynamics, and surgeon preference. The fenestrated lateral tunnel technique predominated in the early half of the study period, while the non-fenestrated extracardiac conduit was used in later years. Fenestrations were typically created using a single 2.8–3 mm punch, irrespective of patient age or weight. Postoperative management followed institutional practices of the cardiothoracic surgery and intensive care unit (ICU) teams rather than a standardized protocol. All patients were extubated in the first 24 hours, with diuretics on postoperative day 1 and titrated based on fluid status and clinical response. Fluid restriction was applied at the care team's discretion. Chest tubes were generally removed when drainage was <2 mL/kg/day, although clinical judgement occasionally warranted deviation. When chylothorax was present, the drainage threshold was reduced to <1 mL/kg/day. Chest radiographs were performed daily, and transthoracic echocardiograms were done 1–2 days postoperatively and at discharge. A low-fat diet was prescribed for 3 months, and patients were routinely seen in the cardiothoracic surgery outpatient clinic within 1 week of discharge.

### Data Collection

Preoperative variables included demographic data (age at Fontan, gender, weight, race), cardiac diagnosis (Hypoplastic left heart syndrome vs other single ventricle heart disease), pre-Fontan catheterization findings (mean PA pressure, mean PA wedge/left atrial pressure, transpulmonary gradient, ventricular end-diastolic pressure, systemic and pulmonary saturations, cardiac index, pulmonary and systemic vascular resistance, Nakata index, presence of aortopulmonary collaterals, and pulmonary artery stenosis) and preoperative cardiac medications.

Operative variables comprised Fontan type (lateral tunnel vs extracardiac conduit), presence of fenestration, cardiopulmonary bypass duration, and aortic cross-clamp time (if applicable).

Postoperative outcomes analyzed were duration of supplemental oxygen, incidence and duration of pleural effusion and chylothorax, chest tube duration, nitric oxide use, need for catheter-based or surgical reintervention prior to discharge, hospital length of stay, discharge cardiac medications (number and dosage of diuretics, use of angiotensin-converting enzyme [ACE] inhibitors and sildenafil), and echocardiographic findings at discharge (ventricular function and Fontan fenestration patency). A Fontan fenestration was considered patent if the mean gradient on Doppler was <8 mm Hg.

Post-discharge variables included detection of pleural effusion on chest x-ray at the first postoperative clinic visit, number and dose of cardiac medications in clinic, and readmission for pleural effusion within 2 months after discharge. Catheter-based or surgical interventions and mortality within 3 months of Fontan discharge were also recorded.

For readmitted patients, their hospital course (including need for ICU admission, chest tube placement, IV diuretics, and catheterization/surgery), hospital length of stay, echocardiographic findings (ventricular function and fenestration patency), and cardiac medications at discharge were reviewed.

Cardiac medication profiles including number, type, and dosage, with emphasis on diuretics were compared between readmission and non-readmission groups at 3 time points: pre-Fontan, discharge, and at maximal therapy. Maximal therapy was defined as the peak intensity regimen used to manage pleural effusion, either at the first postoperative clinic visit or at discharge following readmission, whichever was greater. Metrics included the maximum number of cardiac medications, the maximum number of diuretics, and maximum doses of furosemide, chlorothiazide, spironolactone, and enalapril. Cardiac medications included were aspirin, naproxen, rivaroxaban, sildenafil, captopril, enalapril, atenolol, sotalol, metoprolol, isradapine, furosemide, chlorothiazide, spironolactone, and bumetanide.

### Statistical Analysis

We conducted univariate analyses to compare patients who were readmitted for pleural effusion (case) versus those who were not (control). Continuous variables were expressed as mean with standard deviation (SD) or median with interquartile range (IQR) and analyzed using unequal variance 2-sample t-test or Wilcoxon rank-sum test, respectively, based on the normality of the distributions. Categorical variables were presented as counts with percentages, and Fisher's exact test was employed. Logistic regression using penalized maximum likelihood estimation was performed to assess the association of individual potential predictor variables with readmission. The effect sizes from logistic regression were reported as odds ratios with 95% confidence intervals. Multivariate analysis was not utilized due to the concern of overfitting the model given our small sample size.

## Results

Our cohort comprised 61 single ventricle patients (mean age 42.0 ± 12.4 months) who underwent the Fontan operation. Nine patients (14.7%) were readmitted for significant pleural effusions. Readmitted patients were older at Fontan compared with controls (50.4 ± 13 vs 40.5 ± 11.8 months; *P* = .025). No significant differences were observed between groups in gender, race, weight at Fontan, diagnosis of hypoplastic left heart syndrome (HLHS), pre-Fontan cardiac catheterization findings, and baseline cardiac medication profile (Table 1).

**Table 1.** Demographics, Perioperative Variables Including Pre-Fontan Cardiac Catheterization Hemodynamics and Findings.

Readmission for Pleural Effusion				
	All Patients	Yes	No	*P*-value
	(*N* = 61)	(*N* = 9)	(*N* = 52)	
*Demographics*				
Age at Fontan (mos.)				
Mean ± SD	41.97 ± 12.36	50.44 ± 13.05	40.50 ± 11.75	**.025**3
Gender, *n* (%)				
Male	44 (72.1%)	8 (88.9%)	36 (69.2%)	.422
Weight at Fontan (kg)				
Mean ± SD	14.38 ± 2.92	16.27 ± 3.62	14.06 ± 2.69	.113
Race, *n* (%)				
Asian	3 (4.9%)	0 (0.0%)	3 (5.8%)	.892
Black	6 (9.8%)	1 (11.1%)	5 (9.6%)	
Hispanic	8 (13.1%)	2 (22.2%)	6 (11.5%)	
Hispanic/White	3 (4.9%)	0 (0.0%)	3 (5.8%)	
White	41 (67.2%)	6 (66.7%)	35 (67.3%)	
HLHS, *n* (%)				
HLHS	31 (50.8%)	5 (55.6%)	26 (50.0%)	1.002
*Pre-Fontan Cardiac Catheterization*				
Ao saturation				
Mean ± SD	83.77 ± 4.77	82.67 ± 6.46	83.96 ± 4.47	.583
PA saturation				
Mean ± SD	67.09 ± 6.91	65.56 ± 10.12	67.36 ± 6.28	.623
PV saturation				
Mean ± SD	95.79 ± 5.50	97.00 ± 2.74	95.58 ± 5.84	.403
PA Pressure				
Mean ± SD	11.22 ± 2.47	11.44 ± 2.30	11.18 ± 2.52	.763
PCWP				
Mean ± SD	6.92 ± 2.53	7.22 ± 2.77	6.87 ± 2.51	.733
TPG				
Mean ± SD	4.15 ± 1.63	4.00 ± 1.22	4.17 ± 1.70	.723
CI				
Mean ± SD	3.64 ± 1.25	4.01 ± 1.10	3.57 ± 1.28	.303
PVRI				
Mean ± SD	1.60 ± 0.78	1.71 ± 0.70	1.58 ± 0.80	.63^3^
Nakata index (mm^2^/m^2^)				
Mean ± SD	262.58 ± 98.83	277.78 ± 97.44	259.73 ± 99.85	.62^3^
APCs, *n* (%)				
Yes	17 (27.9%)	1 (11.1%)	16 (30.8%)	.42^2^
PA stenosis, *n* (%)				
Yes	15 (24.6%)	2 (22.2%)	13 (25.0%)	1.00^2^
*Pre-Fontan Medications*				
No. of Cardiac Medications Pre-Fontan				
Median (IQR)	2.00 (1.00, 3.00)	2.00 (2.00, 2.00)	2.00 (1.00, 3.00)	.67^1^
On Diuretics Pre-Fontan *n* (%)				
Yes	13 (21.3%)	2 (22.2%)	11 (21.1%)	1.00^2^
On ACEi Pre-Fontan, *n* (%)				
Yes	36 (59%)	6 (66.7%)	30 (57.7%)	.72^2^

*Note*: Demographics, pre-Fontan catheterization hemodynamics, and perioperative variables by pleural effusion readmission status. Data are presented as mean (standard deviation), median (interquartile range), or *n* (%) as appropriate. *P*-values were calculated using the Wilcoxon rank sum test,^1^ Fisher's exact test,^2^ or unequal variance 2-sample *t*-test,^3^ as appropriate.

Abbreviations: ACEi, angiotensin-converting enzyme inhibitor; Ao, aortic; APCs, aortopulmonary collaterals; CI, cardiac index; HLHS, hypoplastic left heart syndrome; PA, pulmonary artery; PCWP, pulmonary capillary wedge pressure; PV, pulmonary vein; PVRI, pulmonary vascular resistance index; TPG, transpulmonary gradient.

*Intraoperative variables*: These variables—including initial fenestration status, type of Fontan, cardiopulmonary bypass time, and cross-clamp use or duration—were similar between groups.

*Postoperative outcomes*: Readmitted patients had a higher incidence of chylothorax (33.3% [3/9] vs 5.8% [3/52], *P* = .04). No significant differences were observed in oxygen therapy duration, chest tube and pleural effusion duration, nitric oxide use, or length of hospital stay. One readmitted patient required dual-chamber pacemaker implantation for second-degree atrioventricular block on postoperative day 4; 1 control patient with persistent hypoxemia underwent transcatheter coil embolization of a venovenous collateral. At discharge, the number of cardiac medications and diuretics, dosage of diuretics (furosemide, chlorothiazide, and spironolactone), and use of ACE inhibitors or sildenafil were similar between groups. Qualitative ventricular systolic function on echocardiography was also comparable. However, absent or restrictive fenestration on discharge echocardiogram was significantly more frequent in readmitted patients (88.9% [8/9] vs 44.2% [23/52], *P* = .03) (Table 2).

**Table 2.** Intraoperative and Postoperative Variables Including Medications at Discharge.

Readmission for Pleural Effusion				
	All Patients	Yes	No	*P*-Value
	(*N* = 61)	(*N* = 9)	(*N* = 52)	
*Intraoperative*				
Fenestration, *n* (%)	33 (54.1%)	4 (44.4%)	29 (55.8%)	.72^2^
Extracardiac Fontan, *n* (%)	32 (52.5%)	6 (66.7%)	26 (50.0%)	.48^2^
CPB time (min)				
Mean ± SD	108.28 ± 39.72	110.00 ± 50.95	107.98 ± 38.06	.91^3^
Cross-clamp time (min)				
Median (IQR)	60.50 (45.00, 84.00)	54.50 (46.50, 59.00)	63.50 (45.00, 88.00)	.30^1^
Cross-clamp, *n* (%)				
Yes	34 (55.7%)	4 (44.4%)	30 (57.7%)	.49^2^
*Postoperative*				
Oxygen duration (days)				
Median (IQR)	3.00 (2.00, 4.00)	2.00 (1.00, 3.00)	3.00 (2.00, 4.00)	.33^1^
Nitric oxide use, *n* (%)				
Yes	3 (4.9%)	0 (0.0%)	3 (5.8%)	1.00^2^
Duration of pleural effusion while in hospital (days)				
Median (IQR)	2.00 (0.50, 5.00)	2.00 (1.00, 5.00)	2.00 (0.00, 5.00)	.63^1^
Chylothorax, *n* (%)				
Yes	6 (9.8%)	3 (33.3%)	3 (5.8%)	.04^2^
Chest tube postoperative (days)				
Median (IQR)	4.00 (3.00, 5.00)	4.00 (3.00, 6.00)	4.00 (3.00, 5.00)	.51^1^
Length of stay (days)				
Median (IQR)	6.00 (4.00, 9.00)	7.00 (4.00, 9.00)	6.00 (4.50, 8.00)	.78^1^
*At Discharge*				
Absent or restrictive Fontan fenestration on ECHO at DC, *n* (%)	31 (50.8%)	8 (88.9%)	23 (44.2%)	.03^2^
Normal ventricular systolic function on ECHO, *n* (%)	57 (93.4%)	9 (100.0%)	48 (92.3%)	1.00^2^
Number of cardiac medications				
Median (IQR)	4.00 (3.00, 4.00)	3.00 (2.00, 5.00)	4.00 (3.00, 4.00)	.76^1^
Number of diuretic medications				
Median (IQR)	2.00 (1.00, 2.00)	2.00 (1.00, 2.00)	2.00 (1.00, 2.00)	.97^1^
Furosemide dose (mg/kg/day)				
Mean ± SD	3.56 ± 0.86	3.50 ± 1.06	3.57 ± 0.84	.84^3^
Chlorothiazide dose (mg/kg/day)				
Mean ± SD	26.18 ± 8.44	26.50 ± 7.97	26.11 ± 8.70	.92^3^
Spironolactone dose (mg/kg/day)				
Mean ± SD	1.78 ± 0.52	1.97 ± 0.38	1.74 ± 0.55	.43^3^
On ACE-inhibitor, *n* (%)				
Yes	42 (68.9%)	4 (44.4%)	38 (73.1%)	.12^2^
Sildenafil, *n* (%)				
Yes	5 (8.2%)	2 (22.2%)	3 (5.8%)	.15^2^

*Note*: Postoperative variables and discharge medications by pleural effusion readmission status. Data are presented as median (interquartile range), mean (standard deviation), or *n* (%) as appropriate. *P*-values were calculated using the Wilcoxon rank sum test,1 Fisher's exact test,^2^ or unequal variance 2-sample *t*-test.^3^

Abbreviations: ACEi, angiotensin-converting enzyme inhibitor; CPB, cardiopulmonary bypass; DC, discharge; ECHO, echocardiogram; IQR, interquartile range; SD, standard deviation.

*Post-discharge course*: Pleural effusion was detected at the first postoperative clinic visit in all readmitted patients (100%) compared with 13.5% (7/52) of controls (*P* < .01). Medical therapy was commonly intensified in patients with recurrent pleural effusion. No reoperations, catheter interventions, or deaths occurred within 3 months post-discharge.

*Readmission course*: Nine patients were rehospitalized for significant pleural effusion, a median of 7 days (IQR 5, 15) after Fontan discharge. All received intravenous diuretics; 7 were admitted to the PICU with chest tube placement, while 2 patients were managed in the Intermediate Cardiac Care Unit. None of the patients necessitated intubation. Echocardiograms at readmission showed normal ventricular function. Among the 4 patients with initial Fontan fenestration, only 1 demonstrated fenestration patency on readmission; 1 had spontaneous closure prior to initial Fontan discharge, and 2 had restrictive fenestrations both at Fontan discharge and readmission. One patient with chylothorax was treated with total parenteral nutrition, octreotide, and steroids. Median readmission length of stay was 7 days (IQR 5, 9). None required a catheter or surgical intervention during their readmission.

*Cardiac medication profile*: Readmitted patients required a greater number of diuretics at maximal therapy compared with controls (median: 3 vs 2; *P* = .01). Maximum number of cardiac medications and maximum doses of furosemide, chlorothiazide, spironolactone, or enalapril did not differ significantly. Notably, our Fontan patients generally required high doses of furosemide and chlorothiazide, approaching 4 mg/kg/day and 30 mg/kg/day, respectively (Table 3).

**Table 3.** Postdischarge Variables.

Readmission for Pleural Effusion				
	All Patients	Yes	No	*P*-value
	(*N* = 61)	(*N* = 9)	(*N* = 52)	
Detection of pleural effusion at first postoperative clinic visit, *n* (%)	16 (26.2%)	9 (100.0%)	7 (13.5%)	**<** **.** **01** ^2^
Max number of cardiac meds Median (IQR)	4.00 (3.00, 5.00)	4.00 (4.00, 5.00)	4.00 (3.00, 4.00)	.13^1^
Max number of diuretics				
Median (IQR)	2.00 (1.00, 3.00)	3.00 (2.00, 3.00)	2.00 (1.00, 2.00)	**.** **01** ^1^
Max furosemide dose (mg/kg/day)				
Mean ± SD	3.68 ± 0.96	4.06 ± 1.46	3.61 ± 0.85	.40^3^
Max chlorothiazide dose (mg/kg/day)				
Mean ± SD	28.85 ± 8.96	32.20 ± 8.71	27.94 ± 8.98	.28^3^
Max spironolactone dose (mg/kg/day)				
Mean ± SD	1.80 ± 0.61	1.97 ± 0.79	1.73 ± 0.54	.52^3^
Max enalapril dose (mg/kg/day)				
Mean ± SD	0.24 ± 0.13	0.20 ± 0.08	0.25 ± 0.14	.28^3^

*Note*: Post-discharge medication variables and outcomes stratified by pleural effusion readmission status. Data are presented as median (interquartile range), mean (standard deviation), or *n* (%) as appropriate. *P*-values were calculated using the Wilcoxon rank sum test,^1^ Fisher's exact test,^2^ or unequal variance 2-sample *t*-test.^3^

Abbreviations: IQR, interquartile range; Max, maximum; SD, standard deviation.

*Predictors of readmission*: Univariate logistic regression identified older age at Fontan, presence of chylothorax, absent or restrictive Fontan fenestration at discharge, and pleural effusion detected at the first postoperative clinic visit as significant predictors of readmission for pleural effusion (Table 4).

**Table 4.** Risk Factors for Readmission for Pleural Effusion.

	Odds Ratio (OR)	95% Confidence Interval (CI)	*P*-Value
Age at Fontan (months)	1.07	1.01, 1.15	.03
Presence of chylothorax	7.62	1.36, 44.25	.02
Absent or restrictive fenestration at DC	7.12	1.44, 70.26	.01
Detection of pleural effusion at the first postoperative clinic visit	115.26	12.43, +infinity	<.01

*Note*: Univariate analysis of risk factors for readmission due to pleural effusion. Age at Fontan was analyzed as a continuous variable. Odds ratios, confidence intervals, and *P*-values were derived from logistic regression using penalized maximum likelihood estimation.

Abbreviations: CI, confidence interva; DC, discharge; OR, odds ratio.

## Comment

Our study revealed that potential risk factors for unplanned readmissions for post-Fontan pleural effusion included older age at Fontan, development of chylothorax, absent or restrictive Fontan fenestration at discharge, and presence of pleural effusion at the first postoperative clinic visit. Readmitted patients also required more diuretics than those who did not.

Pleural effusions remain a common complication following Fontan palliation, with studies reporting incidence rates of 10%–40%.^5,11,12^ Although there is no consensus on the established risk factors for pleural effusion post-Fontan, several studies have identified potential predictors: elevated mean pulmonary artery pressures,^13^ older age at Fontan,^14^ lower preoperative oxygen saturation, prolonged cardiopulmonary bypass time, presence of post op infection/ surgical timing during respiratory season, prolonged mechanical ventilation,^5,15,16^ impaired ventricular function,^17^ presence of atrioventricular valve insufficiency,^18^ lack of fenestration^19^ and the presence of chylothorax.^20,21^

The impact of Fontan timing on outcomes remains variably reported in the literature. In our study, older age at the time of Fontan was associated with an increased risk of readmission for pleural effusion, with each additional month at Fontan conferring 7% increased odds of readmission. To our knowledge, few studies have directly evaluated the role of Fontan timing as an independent risk factor for pleural effusion-related readmissions. A few studies support our findings. Bezuska et al found shorter chest tube duration in patients undergoing Fontan before 3 years of age.^14^ Cao et al also observed that patients older than 4 years had longer pleural drainage compared with those who were younger.^22^ Conversely, Fontan completion after the age of 10 was associated with increased incidence of prolonged drainage and more postoperative complications.^23^ Although the mechanism by which timing of Fontan completion impacts the risk of pleural effusion remains speculative, recent data suggest that older age at Fontan may be associated with progressive lymphatic abnormalities and increased lung stiffness,^23,24^ both of which could impair pleural fluid resorption and contribute to persistent effusions. In contrast, studies by Gupta et al and Kim et al reported that younger age at Fontan was associated with increased volume of pleural drainage and prolonged pleural effusions, respectively.^5,12^ However, the latter study included patients who underwent Fontan at a younger age (2.8 years) than our cohort (3.5 years). Other investigators have found no significant age difference between patients with and without prolonged pleural effusions.^21^ This inconsistency likely reflects variations in patient selection, surgical approach, staging strategies, and institutional practices. It further highlights the multifactorial nature of postoperative effusions, suggesting that other clinical and physiological variables may have greater influence than age alone.

Nearly 10% of all our patients developed chylothorax, which is consistent with the incidence reported by others.^25^ Among those who were readmitted, one-third had chylothorax compared with those in the non-readmitted cohort. This association is compatible with other investigations which have identified chylothorax as a significant predictor of persistent pleural effusions, often reflected in increased chest tube utilization, delayed chest tube removal, and longer hospital stays.^20,26^ It is proposed that elevated lymphatic afterload is inherent to the Fontan circulation promoting chylous leakage to the pleural space resulting in loss of protein, fat and immunologic factors from the extravasated chyle and compensatory lymphatic production in an already compromised system.^20,27^ Inflammation has also been described as a potentiator for persistent pleural effusions in the context of chylothorax; as inflammation secondary to surgery and cardiopulmonary bypass contribute to capillary leak and lymphatic fluid extravasation.^28^ Other studies describe the consequence of hormonal dysregulation between aldosterone, vasopressin, and atrial natriuretic factor, leading to pleural effusion development post-Fontan.^29,30^ Congenital lymphatic abnormalities have also been observed in patients with single ventricle physiology through prenatal identification using fetal magnetic resonance imaging,^31,32^ possibly highlighting the role of anatomical lymphatic anomalies that predispose single ventricle patients to develop persistent pleural effusions. Although our study did not examine specific risk factors for chylothorax, prior studies have suggested associations with elevated central venous pressure, a systemic right ventricle, fat-containing diets, and lymphatic abnormalities.^33,34^ However, inconsistent findings across the literature highlight a knowledge gap that complicates the prediction of persistent pleural effusions, in part due to the unpredic and multifactorial nature of chylous effusions.^35^

In our study, all re-admitted patients had pleural effusions at the time of their first postoperative clinic visit and eventually required a higher number of diuretic medications (median of 3 diuretics) compared with the non-readmitted group. These observations may reflect differences in post-discharge diuretic management among practitioners and support the idea that closer outpatient monitoring and more aggressive diuretic regimens may be required to prevent the need for rehospitalizations.^36^ In this context, application of a standardized post-Fontan care pathway with structured diuretic management may be beneficial as has been shown by other investigators to reduce pleural effusions, hospital readmissions, length of stay, and overall healthcare costs.^3,37^ Pike et al reported success with a protocol that used triple diuretic therapy beginning with intravenous furosemide on postoperative day 1, transitioning to oral furosemide 3 times daily before discharge, alongside oral chlorothiazide and spironolactone given twice daily.^3^ This regimen continued for 2 weeks after discharge and was tapered based on clinical response. The protocol also included fluid restriction to 80% of maintenance, a low-fat diet, and delayed chest tube removal until drainage was less than 2 mL/kg/day and oral intake was tolerated.^3^ Al-Shawk et al also echoed the importance of early and sustained diuretic use in combination with fluid and dietary management as standard practice in post-Fontan care.^7^ Expanding into the outpatient setting, Cashen et al emphasized the value of structured discharge planning, individualized diuretic regimens, and close monitoring to prevent late effusions and reduce readmissions.^36^ Together, these coordinated strategies represent an evidence-based, multidisciplinary approach to minimizing pleural complications and optimizing recovery in the early Fontan period. Our findings certainly support the strategy of high-dose triple diuretic therapy in the immediate post-Fontan period, especially for those who have other risk factors for recurrent pleural effusion.

In this study, readmitted patients were associated with absent or restrictive fenestration at discharge. Although only 4 out of 9 readmitted patients (44%) had an initial Fontan fenestration, it was an interesting finding that only 1 had a patent fenestration at discharge. A large meta-analysis demonstrated that fenestration was associated with decreased pleural drainage and lower pulmonary artery pressures at the expense of lower oxygen saturations.^38^ A prospective randomized study of standard-risk Fontan patients reported lower chest tube drainage, shorter hospital course, and less postoperative interventions in fenestrated patients.^6^ While in a multicenter registry, univariate analysis showed that although there was no difference in pleural effusion between fenestrated and non-fenestrated groups, the fenestrated group had less incidence of chylothorax.^39^ Conversely, Fiore et al demonstrated higher chest tube drainage in fenestrated HLHS patients but equivalent long-term outcomes regardless of fenestration.^40^ These conflicting findings likely reflect differences in patient populations, institutional practices, and study design. Our study supports consideration of strategies toward fenestration patency in the early post-Fontan period, especially in high-risk patients. Some centers have shown profound effects of transcatheter fenestration dilation^41^ and fenestration creation with stenting^42^ to manage persistent pleural effusion and postoperative low cardiac output syndrome.

The most significant limitation of our study was the small sample size. This is attribu to the single-center nature of the study as well as the inherent rarity of single ventricle heart disease, further emphasizing the importance of multicenter collaboratives and registries to enhance study power and generalizability. Another limitation is our lack of an established, institutionalized protocol for the management of persistent pleural effusions following the Fontan procedure. Lastly, given the retrospective nature of the study, the causal association with the risk factors could not be established.

Despite these limitations, our study offers novel insights into clinical predictors and management strategies aimed at reducing unplanned readmissions for recurrent pleural effusion following Fontan completion—a complication that, to our knowledge, has not been characterized in prior literature. Additionally, our detailed analysis of cardiac medication profiles, including maximal diuretic regimen and dosing, adds granularity to postoperative management considerations. These findings highlight opportunities to optimize care and reduce the burden of repeated hospitalizations—not only through potential healthcare cost savings but also by mitigating patient anxiety/emotional distress and minimizing parental work absences.

We propose that single ventricle patients who had later Fontan timing (ie >4 years), developed chylothorax, had absent/restrictive fenestration at discharge, and had pleural effusion at the first postoperative clinic visit, should have early optimization of diuretic therapy and fluid restriction, closer outpatient monitoring, and consideration of transcatheter fenestration intervention.

Future research should focus on multicenter, prospective studies to better define risk factors for post-Fontan complications and improve the generalizability of the results across diverse populations and institutional practices. These types of collaborations could provide the required statistical power to refine patient selection, optimize perioperative care, and evaluate postoperative management. Furthermore, it may broaden consensus and adoption of standardized post-Fontan management protocols aimed at promoting a more homogenous consistency in care, support improved patient outcomes, and enable more meaningful comparisons across centers.

## Conclusions

Older age at Fontan, chylothorax, absent or restrictive fenestration at discharge, and early detection of reaccumulating pleural effusion were identified as predictors for pleural effusion-related readmissions. These patients required intensified diuretic therapy. Our findings suggest that earlier Fontan timing, aggressive chylothorax management, vigilant outpatient follow-up, and individualized diuretic and fenestration strategies may help reduce post-Fontan complications and readmissions.

## Supplemental Material

sj-pdf-1-pch-10.1177_21501351261418295 - Supplemental material for Unplanned Readmissions due to Pleural Effusion Following the Fontan Operation Supplemental material, sj-pdf-1-pch-10.1177_21501351261418295 for Unplanned Readmissions due to Pleural Effusion Following the Fontan Operation by Jean M. Meneses, Mohan Pankhuri, Jake Beerel, Joseph B. Clark, John L. Myers, Allen R. Kunselman, Akif Ündar and Vincent P. R. Aluquin in World Journal for Pediatric and Congenital Heart Surgery

## Abbreviations

ACEi: angiotensin-converting enzyme inhibitor
AO: aorta
APCs: aortopulmonary collaterals
CI: cardiac index
CPB: cardiopulmonary bypass
HLHS: hypoplastic left heart syndrome
IQR: interquartile range
MAX: maximum
OR: odds ratio
PA: pulmonary artery
PCWP: pulmonary capillary wedge pressure
POST DC: post-discharge
PV: pulmonary vein
PVRI: pulmonary vascular resistance index
SD: standard deviation
TPG: transpulmonary gradient
